# Dispersion of Recovery and Vulnerability to Re-entry in a Model of Human Atrial Tissue With Simulated Diffuse and Focal Patterns of Fibrosis

**DOI:** 10.3389/fphys.2018.01052

**Published:** 2018-08-07

**Authors:** Richard H. Clayton

**Affiliations:** Department of Computer Science, Insigneo Institute for in-silico Medicine, University of Sheffield, Sheffield, United Kingdom

**Keywords:** atrial fibrillation, Courtemanche model, Gaussian random field, fibrosis, computer model, cardiac electrophysiology

## Abstract

Fibrosis in atrial tissue can act as a substrate for persistent atrial fibrillation, and can be focal or diffuse. Regions of fibrosis are associated with slowed or blocked conduction, and several approaches have been used to model these effects. In this study a computational model of 2D atrial tissue was used to investigate how the spatial scale of regions of simulated fibrosis influenced the dispersion of action potential duration (APD) and vulnerability to re-entry in simulated normal human atrial tissue, and human tissue that has undergone remodeling as a result of persistent atrial fibrillation. Electrical activity was simulated in a 10 × 10 cm square 2D domain, with a spatially varying diffusion coefficient as described below. Cellular electrophysiology was represented by the Courtemanche model for human atrial cells, with the model parameters set for normal and remodeled cells. The effect of fibrosis was modeled with a smoothly varying diffusion coefficient, obtained from sampling a Gaussian random field (GRF) with length scales of between 1.25 and 10.0 mm. Twenty samples were drawn from each field, and used to allocate a value of diffusion coefficient between 0.05 and 0.2 mm^2^/ms. Dispersion of APD was assessed in each sample by pacing at a cycle length of 1,000 ms, followed by a premature beat with a coupling interval of 400 ms. Vulnerability to re-entry was assessed with an aggressive pacing protocol with pacing cycle lengths decreasing from 450 to 250 ms in 25 ms intervals for normal tissue and 300–150 ms for remodeled tissue. Simulated fibrosis at smaller spatial scales tended to lengthen APD, increase APD dispersion, and increase vulnerability to sustained re-entry relative to fibrosis at larger spatial scales. This study shows that when fibrosis is represented by smoothly varying tissue diffusion, the spatial scale of fibrosis has important effects on both dispersion of recovery and vulnerability to re-entry.

## 1. Introduction

Although atrial fibrillation (AF) is a common cardiac arrhythmia, the mechanisms that initiate and sustain it are complex and remain incompletely understood. Circulating waves of electrical activation and recovery, often called re-entrant waves or rotors, are believed to sustain AF (Nattel, 2002). The anatomy of the human atria is complex, and structural heterogeneity is considered to be an important substrate for atrial arrhythmias (Haissaguerre et al., 2016; Zhao et al., 2017). Fibrosis is an important component of heterogeneity (Csepe et al., 2017), although the role played by regions of fibrosis in anchoring rotors remains controversial (Nguyen et al., 2014; Trayanova et al., 2014; Sohns et al., 2017). Magnetic resonance imaging of the heart using delayed enhancement enables regions of fibrosis to be imaged directly; this approach has been used to reveal the different spatial scales of fibrosis that are present (Tanaka et al., 2007), to stratify patients and guide intervention (Akoum et al., 2012), and to demonstrate an association between the presence of fibrosis and recurrent atrial arrhythmias in patients (Marrouche et al., 2014).

Fibrosis is hard to study in experimental preparations because it is difficult to control the extent of fibrosis and the size of individual lesions, and so computational models of cardiac cell and tissue electrophysiology have been used to examine how regions of simulated fibrosis affect activation and recovery in cardiac tissue, as well as vulnerability to arrhythmias (Trayanova et al., 2014). When regions of fibrosis are represented as inexcitable obstacles, diffuse fibrosis can act to slow activation and to destabilize re-entry in simulated ventricular tissue (Ten Tusscher and Panfilov, 2007). This approach was extended by representing fibroblasts as coupled elements with a fixed resting potential instead of inexcitable regions, and similar effects were found (Majumder et al., 2012). A more recent study has shown that the strength of coupling between normal tissue and simulated fibroblasts as well as the fibroblast resting potential can modify the tissue response (Sridhar et al., 2017). The density of simulated fibrosis was identified as important in a study that used detailed anatomical models of infarcted rabbit ventricles; intermediate densities increased susceptibility to arrhythmia whereas high densities suppressed arrhythmia (McDowell et al., 2011). In patient specific models of atrial fibrosis, the particular shape and configuration of fibrotic regions has been observed to have an important effect on rotor behavior (McDowell et al., 2015; Morgan et al., 2016). The effect of spatial scale of fibrosis has also been investigated in simulations where fibrosis was represented as regions of uncoupled and inexcitable ventricular tissue; this study found that an increasing amount of fibrosis and increasing spatial scale both acted to increase vulnerability to arrhythmia (Kazbanov et al., 2016). In simulated atrial tissue, the electrotonic effect of regions of fibrosis was studied using a coupled and detailed fibroblast model to demonstrate that the effect of fibroblasts could underlie the complex fractionated electrograms often seen in persistent atrial fibrillation (Ashihara et al., 2012).

The role of heterogeneous tissue properties in increasing vulnerability to atrial arrhythmias is well-established (Allessie et al., 1976; Vigmond et al., 2004; Haissaguerre et al., 2016; Gokhale et al., 2017), but the role played by the size or length scale of fibrosed regions is not well-understood. Some simulation studies have represented heterogeneity by a chequerboard, where the size of the squares can be modified to control the length scale (Xie et al., 2001; Clayton and Holden, 2005; Kazbanov et al., 2016). Other studies have based the configuration of fibrosis on experimental (Tanaka et al., 2007; Engelman et al., 2010) or clinical (Morgan et al., 2016; Zahid et al., 2016) images. However the sharp boundaries imposed by these approaches where tissue is designated as either fibrosed or normal may not represent the smooth changes that might be expected in cardiac tissue.

One technique for representing smoothly varying quantities is a correlated random field, where the overall properties of the field are random, but neighboring regions are correlated so that the field varies smoothly according to a correlation length. One class of correlated random field is a Gaussian random field (GRF) with exponential covariance, which is specified by a mean, a variance, and a correlation length. In 2-dimensions (2D), a GRF can be sampled to produce a series of random fields. In each sample, the values of the field will be correlated in both principal directions, and will be normally distributed. However the configuration of fluctuations in the field will vary from sample to sample. This approach has been used in models of groundwater flow with varying hydraulic conductivity (Meerschaert et al., 2013), and the heterogenous spread of disease (Baptista et al., 2016).

The aim of the present study was to use a GRF to generate samples of 2D atrial tissue with fibrosis represented as smoothly varying diffusion, and to investigate the effect of heterogeneous diffusion at different length scales on electrical activation, recovery, action potential duration (APD), vulnerability to re-entry, and dynamics of re-entry.

## 2. Methods

### 2.1. Overall approach

Electrical activation and recovery were simulated in a 2D sheet of atrial tissue, to enable the effect of cell and tissue electrophysiology to be examined without the additional complexity of anatomical structure. To represent the effect of fibrosis, the diffusion coefficient was set to vary smoothly from a low value representing regions of fibrosis, up to higher levels representing normal excitable tissue. The length scale of fluctuations in diffusion coefficient was varied by taking samples from GRFs with different correlation lengths. The effects of heterogeneities in diffusion at different length scales were examined by simulating normal beats during pacing at a steady cycle length, a single premature stimulus, vulnerability to re-entry during rapid decremental pacing, and behavior of an imposed spiral wave. These simulations were run using model parameters set to represent normal human atrial cells, and cells that have undergone remodeling as a consequence of persistent AF.

### 2.2. Cellular electrophysiology model and implementation

Cardiac cellular electrophysiology was modeled using the Courtemanche-Ramirez-Nattel (CRN) model of human atrial cells (Courtemanche et al., 1998). To avoid instability resulting from drift in intracellular ion concentrations (Cherry and Evans, 2008; Wilhelms et al., 2012), [Na^+^]_*i*_ and [K^+^]_*i*_ were fixed at their default initial values of 11.17 and 139.00 mM, respectively. The CRN model was implemented in C, based on code automatically generated from the CellML repository (www.cellml.org).

Two variants of the cell model were used in this study. The first variant, denoted *CRNnormal*, used model parameters as specified in the original paper (Courtemanche et al., 1998) and curated in CellML. This variant produces an action potential with a pronounced spike and dome, and an APD of around 300 ms. The second variant, denoted *CRNremodelled*, simulated the effects of cellular remodeling due to persistent AF. In *CRNremodelled*, the maximum conductance of *I*_*to*_ and *I*_*Ca, L*_ were decreased by 65% (i.e., multiplied by 0.35), *I*_*K, ur*_ was decreased by 49%, and the maximum conductance of *I*_*K*1_ was increased by 110%. These changes are based on those described in a previous study (Wilhelms et al., 2012), and produced a shortened action potential with a less prominent spine and dome that was consistent with experimental observations in cells from remodeled hearts (Workman et al., 2001). Initial conditions for gating and other variables in each simulation were set by pacing each variant at a cycle length of 1,000 ms for 40 beats.

### 2.3. Tissue model and gaussian random field

Cardiac tissue was simulated using the monodomain model (Clayton et al., 2011), where tissue conductivity can be represented as a diffusion coefficient. Electrical activation and recovery were studied in a 2D sheet of tissue, with 400 × 400 grid points representing dimensions of 10 × 10 cm, isotropic diffusion, and no-flux boundary conditions at each edge. The monodomain model was solved using a finite difference method, with a space step of 0.25 mm, and an adaptive time step that ranged between 0.1 and 0.01 ms (Rush and Larsen, 1978; Qu, 1999). With a uniform diffusion coefficient of 0.2 mm^2^ms^−1^, this configuration yielded a plane wave conduction velocity of 0.695 mm ms^−1^ for the *CRNnormal* variant, which is at the lower end of the range of conduction velocity observed in human atria (Weber et al., 2011).

Gaussian random fields (GRFs) can represent a smoothly varying field in space, and are composed of a mean and a covariance. The covariance function describes correlations between the value of the field at a single point and within its neighborhood. A squared exponential covariance function includes a correlation length δ, which can be used to vary the length scale of fluctuations in the field.

(1) $$\begin{array}{l} Cov\left(x,y\right)=exp\left\{\frac{{\left(\left|x-y\right|\right)}^{2}}{\delta^{2}}\right\} \end{array}$$

**Figure 1:**
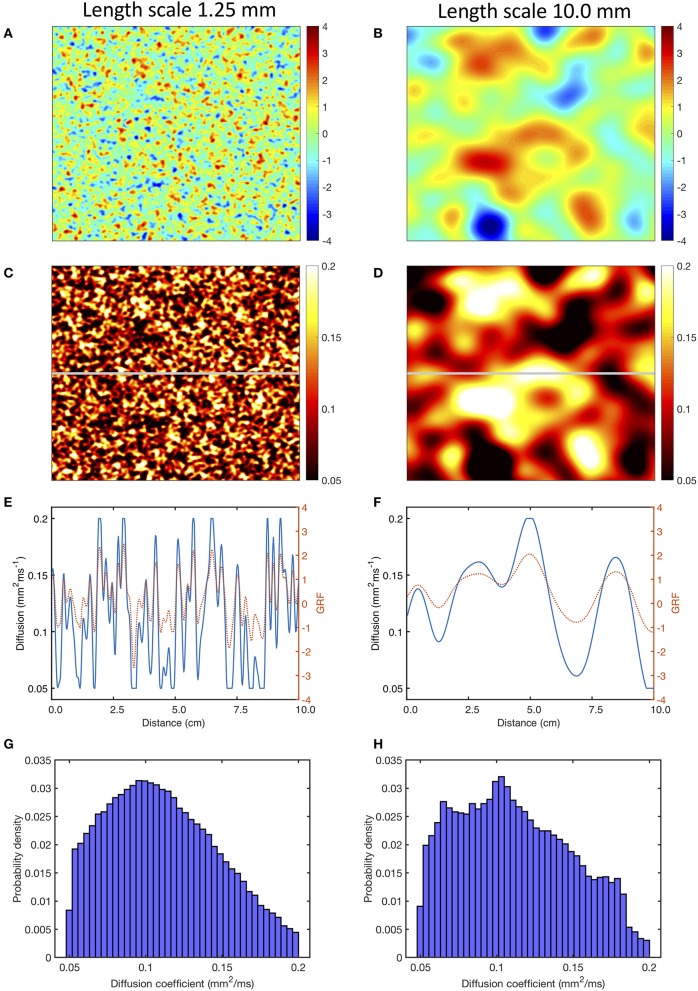
Use of a Gaussian random field (GRF) to generate heterogenous and smoothly varying diffusion. Panels **(A,B)** show samples of GRF with mean of 0 and variance of 1, with spatial length scales of 1.25 and 10 mm, respectively. Panels **(C,D)** corresponding diffusion fields, calculated as described in the main text, with a cut-off of 0.05 and 0.2 mm^2^ms^−1^. Panels **(E,F)** show the profile of GRF (red, dotted) and diffusion coefficient (blue) across the middle row of each sample indicated by the horizontal gray line in **(C,D)**. Lower panels **(G,H)** show the distribution of the diffusion fields shown in **(C,D)**.

Stationary GRFs with mean of 0, variance of 1.0, and an exponential covariance function were generated with length scales of 1.25, 2.50, 5.0, and 10.0 mm using the method of circulant embedding, which is detailed in Kroese and Botev (2015). Samples of these GRFs with a length scale of 1.25 and 10 mm are illustrated in Figures 1A,B. To translate these fields into a diffusion field, the raw GRF was offset by 2, and multiplied by 0.05. GRF raw values of −1, 0, and +1 therefore translated to diffusion coefficients of 0.05, 0.1, and 0.15 mm^2^ms^−1^, respectively. The value of the diffusion coefficient was capped at 0.2 mm^2^ms^−1^.

Regions with a diffusion coefficient less than 0.05 mm^2^ms^−1^ were designated areas of fibrosis, and were set to be coupled but inexcitable, with a diffusion coefficient of 0.05 mm^2^ms^−1^. Inexcitability was imposed by setting the membrane current term in the monodomain equation to zero, while retaining the term describing voltage diffusion.

Figures 1C, D show the diffusion fields corresponding to Figures 1A,B, and the profile of the raw GRF as well as the diffusion coefficient for the central row of each sample are plotted in Figures 1E,F. The lower panel of Figures 1 shows the distribution of diffusion coefficient in the samples of the GRF at each length scale. In these distributions the median is close to 0.1 mm^2^ms^−1^ as expected, and the effect of truncation at 0.05 and 0.2 mm^2^ms^−1^ can be seen.

Since the configuration of fibrotic regions would be expected to affect simulated tissue behavior, 20 samples of the GRF were obtained at each of the four length scales. The procedure described above was used to generate a set of 20 diffusion fields, and these were then used for simulations with the different pacing protocols described in the next section. Multiple samples enabled the effect of length scale to be assessed independent of the specific configuration of a single sample relative to pacing sites. Each sample was then used to simulate the different pacing protocols specified below, with both *CRNnormal* and *CRNremodelled* variants.

To compare the effect of the smooth variations in diffusion coefficient provided by the GRF with abrupt changes, a further set of diffusion fields were generated. For each length scale, a copy was made of each of the 20 diffusion fields obtained by sampling the GRF. These copies were then further processed so that they contained only two values of diffusion coefficient, with an abrupt change between the two. In the fibrotic regions, the diffusion coefficient of 0.05 mm^2^ms^−1^ was retained, but elsewhere it was set to 0.2 mm^2^ms^−1^.

Within the 20 diffusion fields generated at each length scale, the average proportion of simulated fibrotic tissue (i.e., locations where the diffusion coefficient was 0.05 mm^2^ms^−1^, and the cell model was set to be ineexcitable) was 18.7, 18.0, 19.1, and 17.0% at length scales of 1.25, 2.50, 5.0, and 10.0 mm, respectively. The size and configuration of these regions was different in each of the 20 samples. At a length scale of 10 mm, the proportion of simulated fibrotic tissue ranged between 4.9 and 25.1% across the 20 samples. With a length scale of 1.25 mm, which was a smaller length scale relative to the size of the simulated tissue, the range was 17.9–19.6%.

### 2.4. Pacing protocols

#### 2.4.1. S1S2 pacing

Activation and recovery of normal and remodeled tissue were studied with regular pacing for three beats at a cycle length of 1,000 ms followed by a premature stimulus with a coupling interval of 400 ms. Stimuli were delivered with a current injection of −2000 pA for 1 ms in a 2.5 × 2.5 mm square region in the lower left hand corner.

#### 2.4.2. Decremental pacing

The response of the each sample of the heterogenous tissue to rapid decremental pacing was examined by pacing from a circular region in the centre of the tissue with a with radius of 6.25 mm. Ten pacing stimuli with a current of −2,000 pA and duration of 1 ms were delivered with an initial coupling interval of 450 ms for the *CRNnormal* variant and 300 ms for the *CRNremodelled* variant, decreasing by 25 ms with each successive stimulus to a final coupling interval of 250 ms for normal tissue and 150 ms for remodeled tissue (Zahid et al., 2016).

#### 2.4.3. Dynamics of re-entry

The dynamic behavior of re-entry in each sample of heterogeneous tissue was studied by imposing an Archimedean spiral on the model as an initial condition using the phase distribution method (Biktashev and Holden, 1998).

## 3. Results

**Figure 2:**
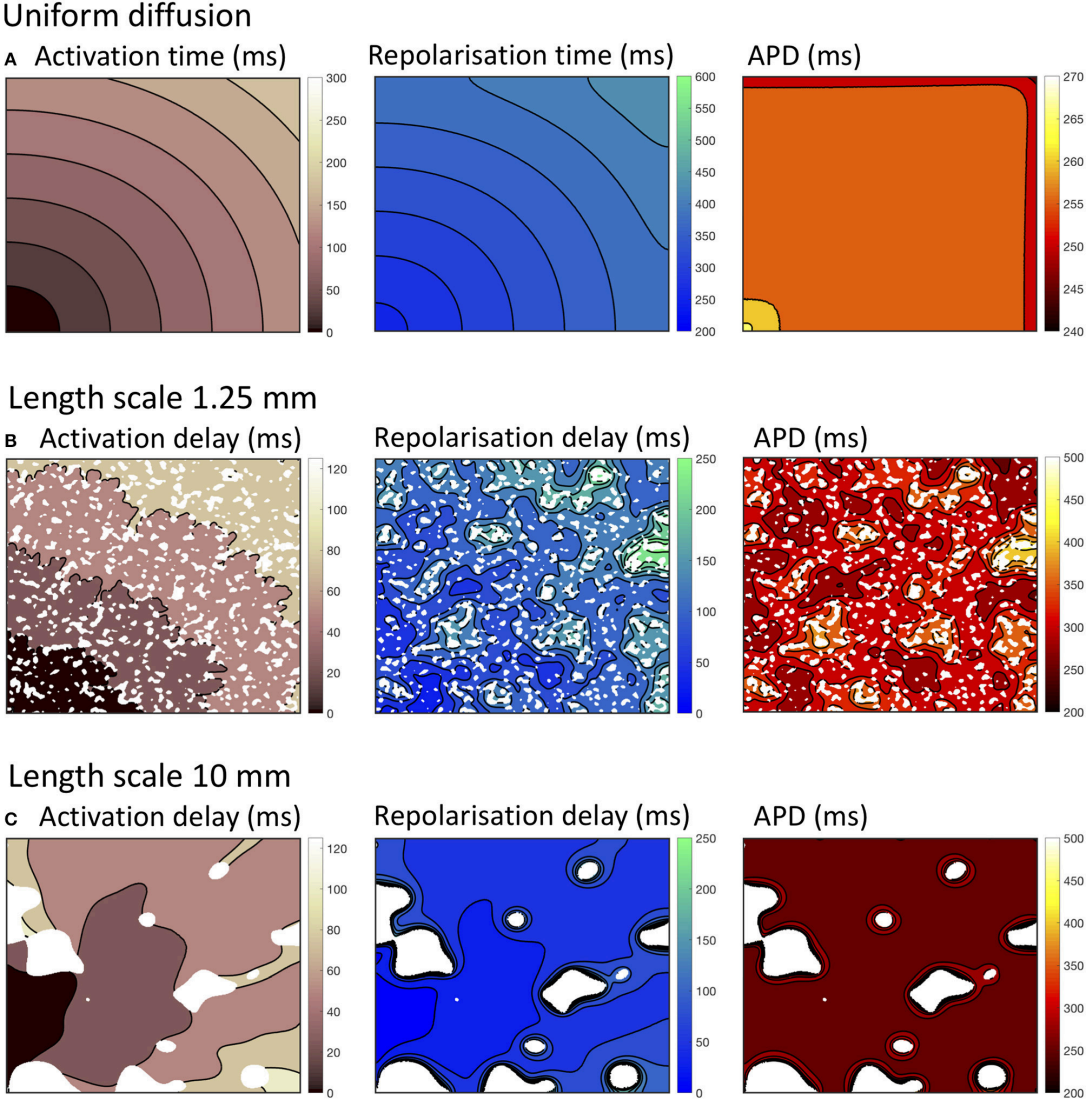
Activation, recovery and action potential duration (APD) in simulations with uniform diffusion **(A)**, heterogeneous diffusion on a length scale of 1.25 mm **(B)**, and a length scale of 10.0 mm **(C)**. The delayed activation and repolarization shown in **(B,C)** are relative to the activation and repolarization in uniform tissue shown in **(A)**. All isochrone contours are at 25 ms intervals except for APD with uniform diffusion, where the contours are at 5 ms intervals.

Examples of the effect of heterogenous diffusion with a length scale of 1.25 mm and 10.0 mm on activation, repolarization, and APD are shown in Figures 2B,C, compared to results for simulated tissue with a uniform diffusion coefficient of 0.2 mm^2^ms^−1^ shown in Figure 2A. In each of these simulations, activation and recovery times were determined from excursions of the local action potential above a threshold of −73.0 mV, which corresponds approximately to the voltage at which repolarization is 90% complete in the CRN model. APD was the difference between repolarization time and activation time. Activation time, repolarization time, and APD were not calculated for regions of fibrotic tissue with diffusion set to 0.05 mm^2^ms^−1^, and these are indicated as white regions in Figures 2B,C.

With uniform diffusion, the activation isochrones were smooth, and recovery isochrones were modified by the boundary resulting in a slightly prolonged APD close to the stimulus site, and a slightly shortened APD on the top and right hand boundary (Cherry and Fenton, 2011). In this configuration, the conduction velocity was 0.507 mm ms^−1^ for the *CRNnormal* and 0.489 mm ms^−1^ *CRNremodelled* variants.

Heterogeneous diffusion with a length scale of 1.25 mm delayed activation by up to 120 ms, and recovery by up to 250 ms. As a result, conduction velocity was lower and APD was prolonged in regions of delayed recovery (Figure 2B). Heterogeneous diffusion with a length scale of 10 mm resulted in a similar delay of activation, but with smoother activation isochrones and a more tortuous activation sequence. Recovery was not prolonged to the same extent, and APD was only prolonged slightly compared to simulations with uniform diffusion (Figure 2C).

**Figure 3:**
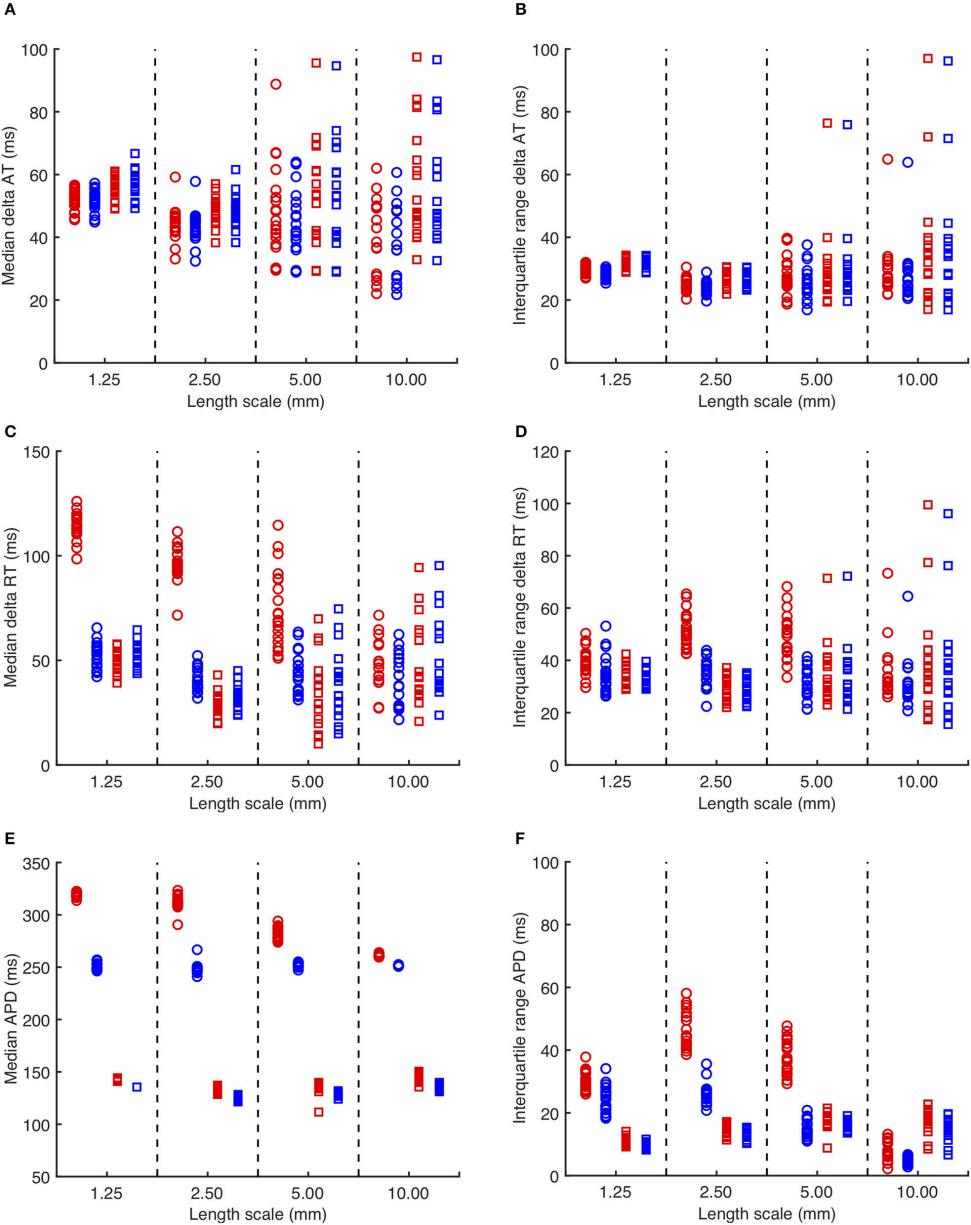
**(A)** Median delay in activation time for each simulation with heterogeneous diffusion compared to uniform diffusion. Red symbols indicate activation following the third S1 stimulus, and blue symbols the S2 stimulus. Circles denote *CRNnormal*, and squares *CRNremodelled*. **(B)** Corresponding interquartile range of activation time delay. **(C,D)** Median and interquartile range of recovery time delay. **(E,F)** median and interquartile range of APD.

The overall effects on activation, recovery, and APD for all of the simulations are shown in Figure 3. In 14/160 simulations with smoothly varying diffusion, either S1 and S2 stimuli (9) or S2 stimuli only (5) were blocked by a region of simulated fibrosis close to the stimulus site at the lower left hand corner. Data from these simulations are not plotted. Activation delays relative to simulations with uniform diffusion were broadly similar for S1 and S2 stimuli, and for *CRNnormal* and *CRNremodelled* cell model variants. This finding reflects the reduced conduction velocity relative to uniform diffusion, resulting from the lower average diffusion in the heterogeneous simulations. The spread of both median and interquartile range activation delay increased somewhat with increasing length scale. This was attributed to different configurations of low diffusion regions having a greater effect on activation pattern at larger length scales. Figure 2C, for example, shows activation that proceeds upwards along one edge before propagating from left to right.

A further set of simulations was conducted using diffusion fields where the smoothly varying diffusion obtained from the GRF was replaced by an abrupt transition from a diffusion coefficient of 0.05 mm^2^ms^−1^ in fibrotic regions to a diffusion coefficient of 0.2mm^2^ms^−1^ elsewhere. A modified version of Figure 3 with these results superimposed is included as Supplementary Figure A. The delay in activation time compared to uniform diffusion was much smaller, but otherwise these simulations showed a broadly similar pattern of behavior.

Delay in recovery was greatest for S1 beats in simulations with the *CRNnormal* variant and a length scale between 1.25 and 5.0 mm. The interquartile range of recovery delay was also greatest for simulations with the *CRNnormal* variant and length scales of 2.5 and 5.0 mm. These delays produced the longest median APD for S1 beats in simulations with the *CRNnormal* variant and a length scale between 1.25 and 5.0 mm, and the greatest APD dispersion for length scales of 2.5 and 5.0 mm.

**Figure 4:**
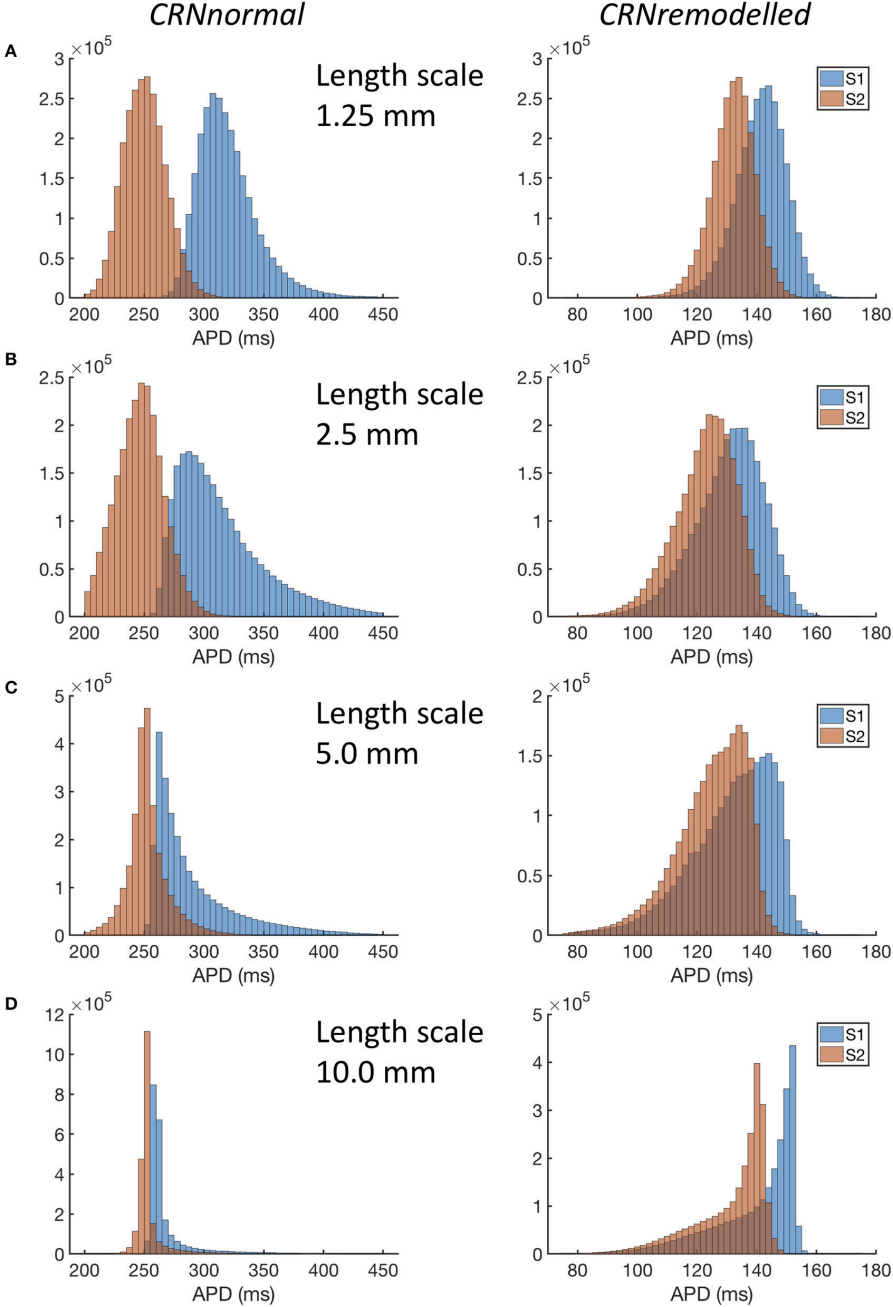
Distribution of APD in simulations with *CRNnormal* (left) and *CRNremodelled* variants, at length scales of 1.25 mm through to 10.0 mm **(A–D)**. Red bars denote APD following third S1 stimulus, and blue bars APD following S2 stimulus.

A more detailed view of the findings is given in Figure 4, where distributions of APD across all 20 simulations in each category are shown. For simulations with the *CRNnormal* variant, the distribution of APD became much narrower as length scale increased. The median and symmetry of APD distribution for the S2 beat was similar at all length scales, whereas the distribution for the S1 beat was positively skewed toward longer APD, especially with a length scale of 2.5 and 5.0 mm. In contrast, with the *CRNremodelled* variant, the distributions for both S1 and S2 beats became negatively skewed toward shorter APD at long length scales.

**Figure 5:**
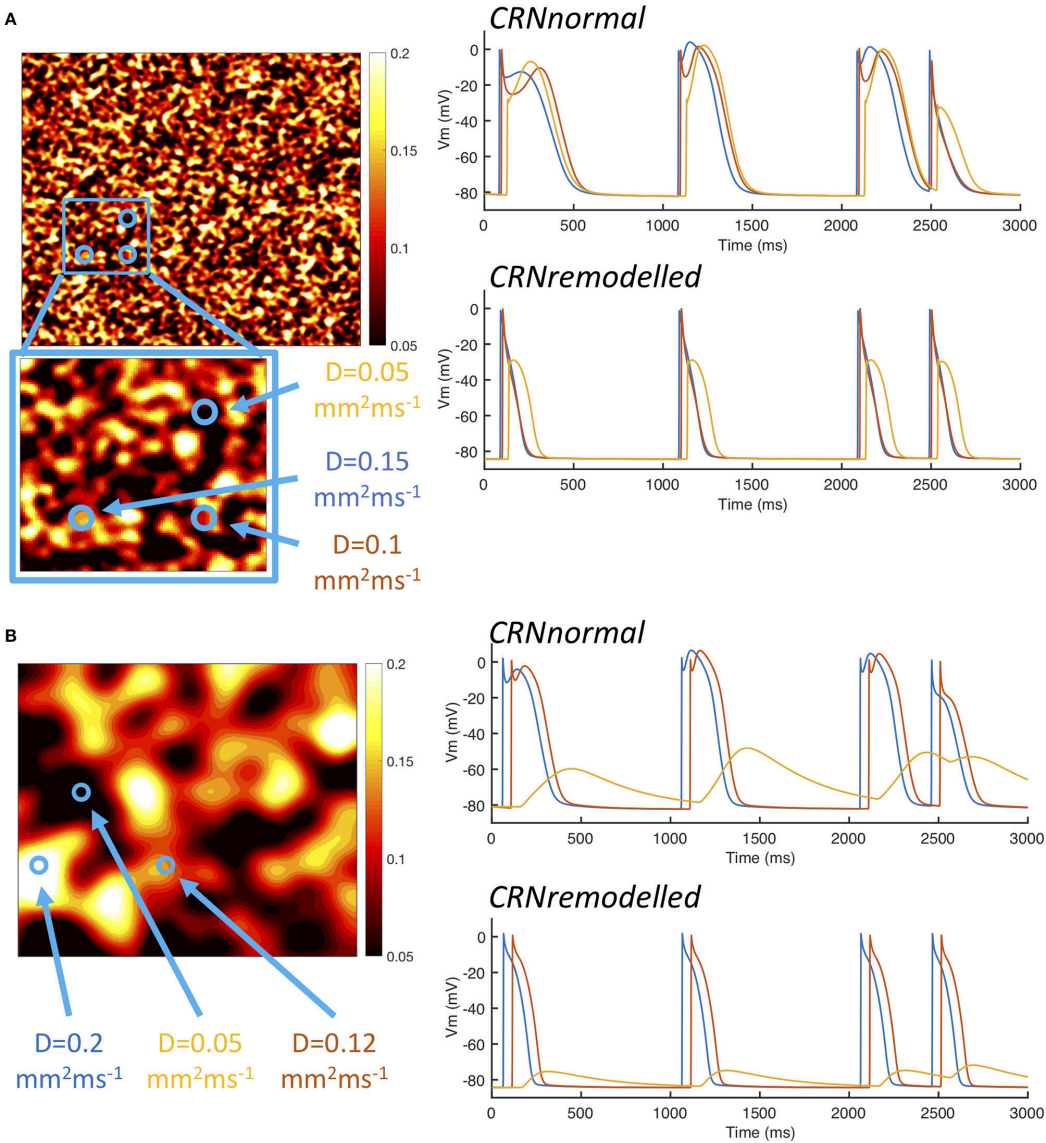
Example action potential shapes from different regions. **(A)** Left panel shows sample of heterogenous diffusion coefficient with length scale of 1.25 mm, inset shows an enlargement of square region outlined in blue. Circles denote three points at which action potential time series were recorded as shown in the right hand panels. Upper panel shows action potential time series with *CRNnormal* variant and lower panel shows *CRNremodelled*. Line colors correspond to point 1 (red), point 2 (blue), and point 3 (yellow). Panel **(B)** shows corresponding plots for sample of heterogenous diffusion with length scale of 10 mm.

A possible explanation for these observations is explored in Figure 5, which shows examples of heterogeneous diffusion at length scale of 1.25 mm (Figure 5A) and 10 mm (Figure 5B), together with voltage time series recorded from locations with diffusion coefficients of 0.2, 0.1, and 0.05 mm^2^ms^−1^. In the model of fibrosis chosen for the present study, regions with a diffusion coefficient of 0.05 mm^2^ms^−1^ were deemed diffusively coupled but inexcitable. These regions could act as a current sink during activation, and as either a current source or a current sink during recovery. The magnitude of this effect was modulated by the length scale of heterogeneity. In Figure 5A, the voltage time series obtained from the inexcitable region with diffusion coefficient of 0.05 mm^2^ms^−1^ (shown in yellow) had a sharp upstroke, and an amplitude and recovery that were comparable in slope to the other time series shown. In contrast, the corresponding voltage time series shown for a length scale of 10 mm in Figure 5B had a much slower upstroke and downstroke, as well as a lower amplitude. For both length scales the shorter APD associated with the *CRNremodelled* variant produced a lower amplitude deflection in the inexcitable regions.

The interaction of local APD, heterogenous diffusion, and the length scale of heterogeneities therefore appears to be complex, and this is emphasized by the range of action potential shapes seen in Figure 5. Heterogenous diffusion acted to delay activation because inexcitable regions act as a current sink, slowing the action potential upstroke, and the delay appeared to be broadly independent of length scale. However, during the action potential, the voltage of inexitable regions increased from a resting value, by an amount that depended on both the length scale of heterogeneity and APD. A greater length scale combined with the shorter APD of the *CRNremodelled* variant resulted in a lower amplitude excursion. The lower amplitude excursion in the *CRNremodelled* variant in turn shortened the recovery of neighboring excitable regions by acting as a current sink. At longer length scales, these neighboring regions were smaller in extent, which could explain in the skewed distribution toward shorter APD seen in the right hand panels of Figure 4, which became more marked at longer length scales. In contrast, the larger amplitude voltage excursion in inexcitable regions produced by the longer APD of the *CRNnormal* variant could act as a current source during recovery, acting to prolong APD, an effect that would have had more impact on neighboring regions. This could explain the skew toward longer APD values seen in the left hand panels of Figure 4.

**Figure 6:**
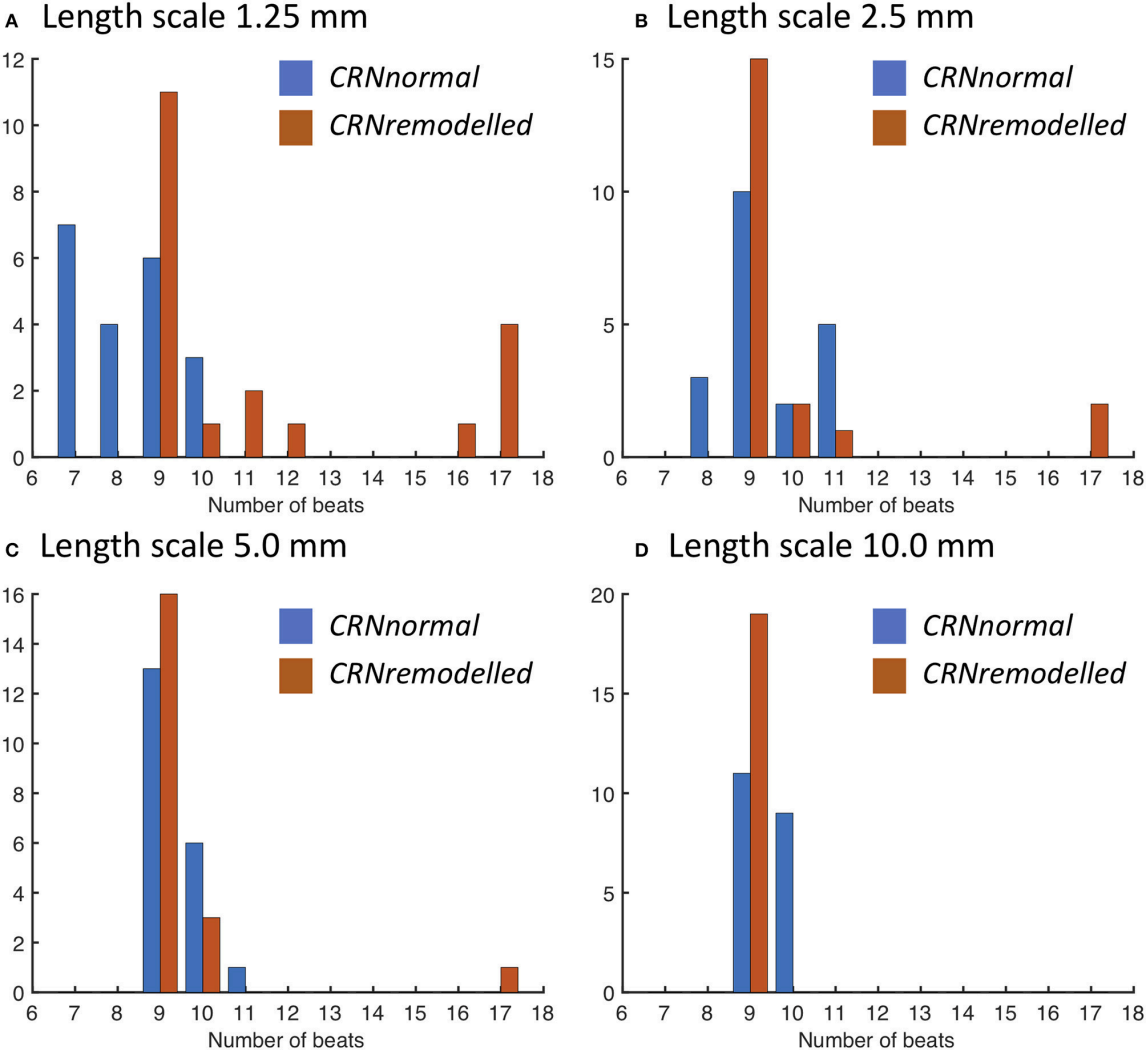
Number of action potentials elicited during decremental pacing at the centre of simulated tissue with heterogeneous diffusion at length scales of **(A)** 1.25, **(B)** 2.5, **(C)** 5.0, and **(D)** 10.0 mm (see text for details). Blue bars denote simulations with the *CRNnormal* variant, and red bars simulations with the *CRNremodelled* variant.

The response of this complex tissue model to decremental pacing with 9 stimuli is illustrated in Figure 6, which shows results from each group of 20 simulations. Additional action potentials after the end of the pacing sequence, or sustained re-entry (deemed to be more than 15 beats), were observed in only a minority of the simulations. For simulations with the *CRNnormal* variant, some configurations of heterogeneity resulted in one or more of the pacing stimuli being blocked, and so the number of beats recorded was less than 9. However, this effect was not observed with the *CRNremodelled* variant, which could be attributed to the greater prolongation of APD in heterogenous tissue with the *CRNnormal* variant described above. Overall, the prevalence of additional beats and re-entry was greater with the *CRNremodelled* variant, and with shorter length scales. Additional simulations were run using diffusion fields where the smoothly varying diffusion obtained from the GRF was replaced by an abrupt transition, and the maximum number of beats elicited was 10. These data are included as Supplementary Figure B.

**Figure 7:**
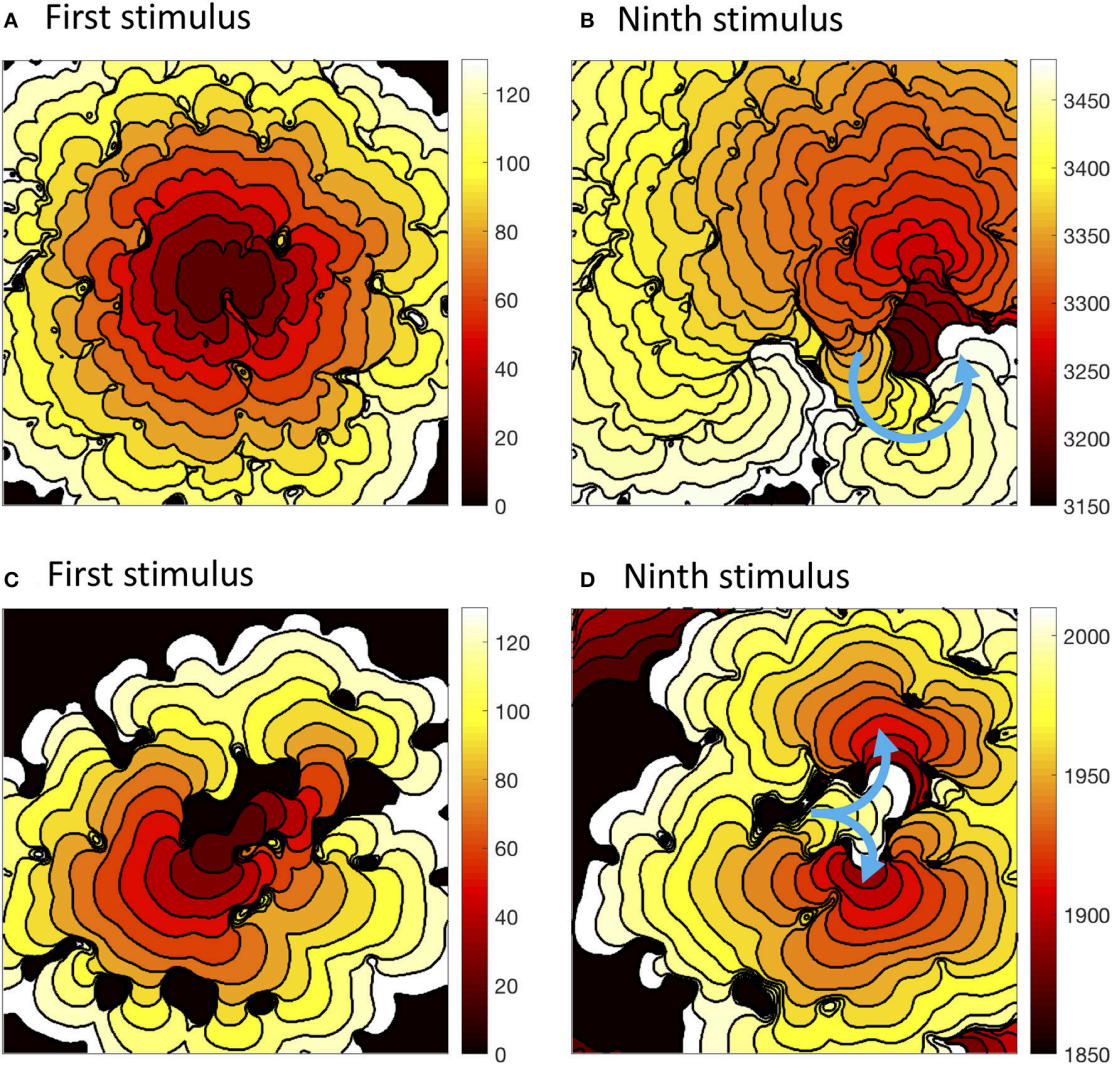
Activation isochrones at 10 ms intervals during decremental pacing in simulations with the *CRNnormal* variant and heterogenous diffusion at a length scale of 2.5 mm with the *CRNnormal* variant **(A,B)** and 5.0 mm with the *CRNremodelled* variant **(C,D)**. Left hand panels show activation following the first stimulus, and right hand panels activation following the ninth stimulus. Blue arrows indicate the subsequent activation pathway for spiral re-entry **(B)**, and figure of eight re-entry **(D)**.

The precise configuration of regions with lower diffusion was important for eliciting re-entry, and this is illustrated in Figure 7. The first activation sequence in Figure 7A shows a lobe of delayed activation running down from the centre, and one side of this lobe provided the activation pathway for re-entry seen in Figure 7B. The narrow activation pathway running toward the bottom right from the centre in Figure 7C became blocked during decremental pacing, and provided a pathway for re-entry following the final stimulus in Figure 7D. Movies of these simulations are available as Videos 1, 2, and an additional example of sustained re-entry in a simulation with a length scale of 2.5 mm and the *CRNremodelled* model variant is illustrated in Video 3.

**Figure 8:**
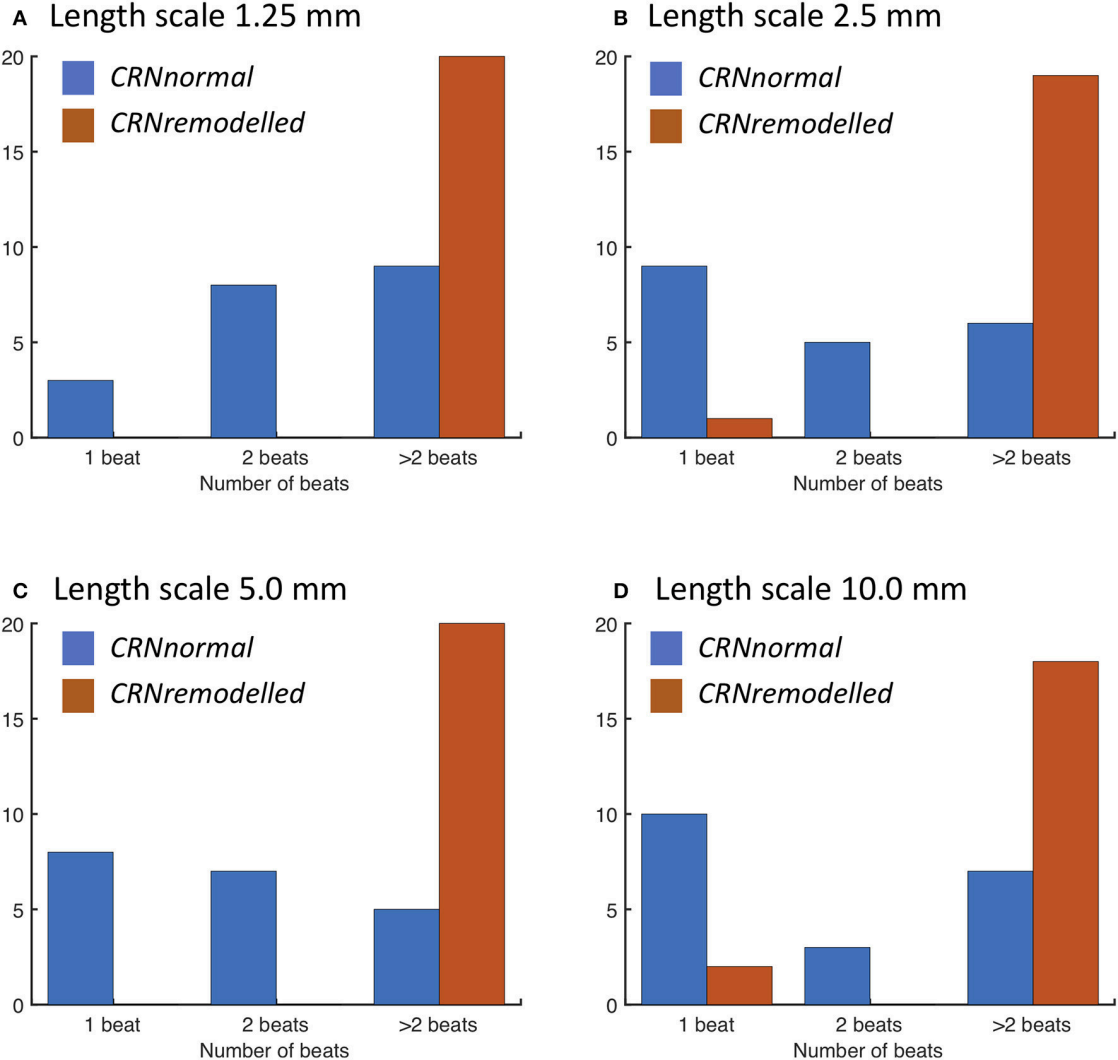
Number of re-entry cycles following initiation with spiral wave with heterogeneous diffusion at length scales of **(A)** 1.25, **(B)** 2.5, **(C)** 5.0, and **(D)** 10.0 mm (see text for details). Blue bars denote simulations with the *CRNnormal* variant, and red bars simulations with the *CRNremodelled* variant.

When a spiral wave was imposed as an initial condition, the configuration of heterogeneities again played a similar role. The number of beats resulting from spiral wave initiation are shown for each length scale in Figure 8. The majority of simulations with the *CRNremodelled* variant resulted in sustained re-entry of more than 2 beats, whereas simulations with the *CRNnormal* variant tended to result in non-sustained re-entry (see Video 4). The length scale of heterogeneity did not play a major role, although there was a slight tendency for less sustained re-entry at larger length scales.

**Figure 9:**
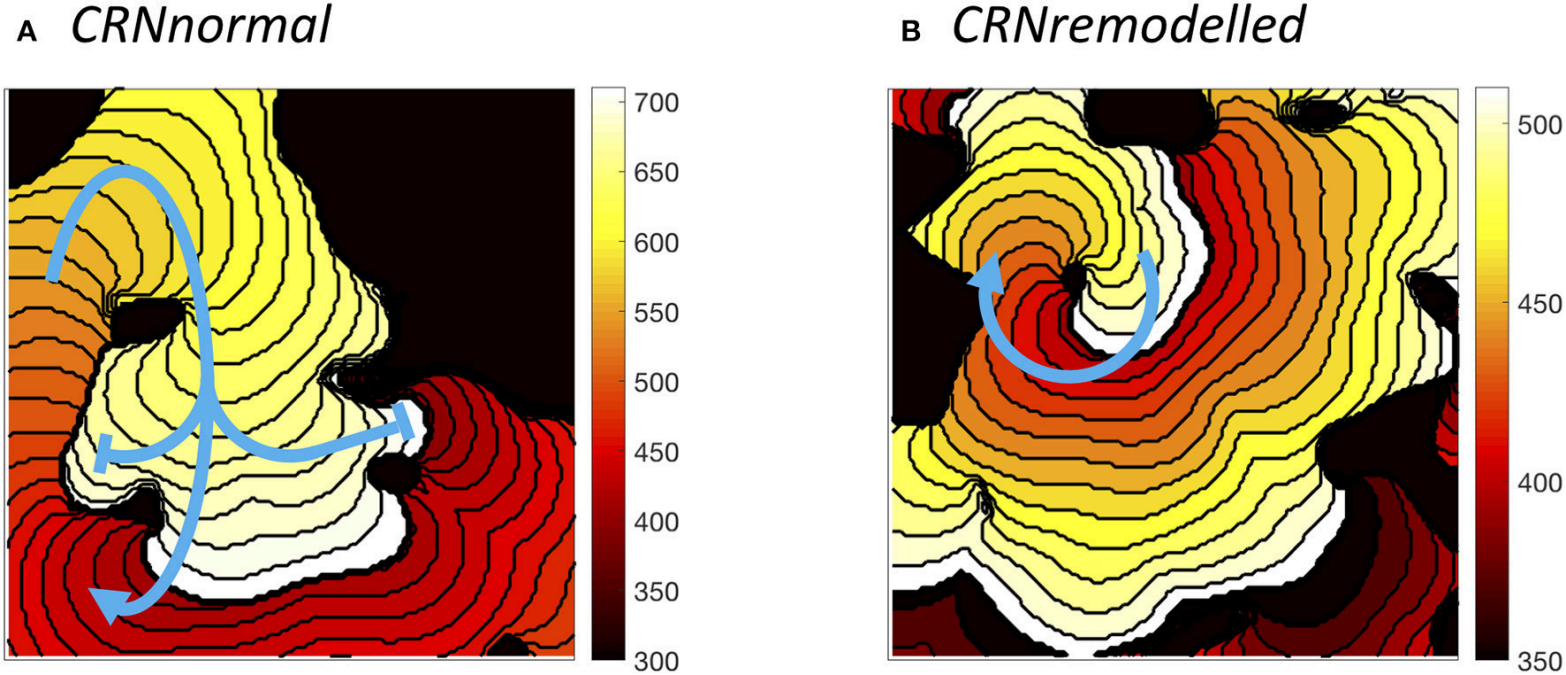
Examples of sustained re-entry elicited by spiral wave initiation with the same pattern of heterogenous diffusion at a length scale of 10 mm. **(A)** re-entry pathway with period of around 310 ms in simulated tissue with *CRNnormal* variant. **(B)** more compact re-entry pathway with much shorter period of around 130 ms in simulated tissue with *CRNremodelled* variant. Activation isochrones spaced at 10 ms intervals.

The wavelength (minimum APD × conduction velocity) is an important tissue property, and excitable tissue must be large enough to accommodate the wavelength in order to sustain re-entry. Heterogeneous diffusion modulates wavelength by slowing conduction velocity (delaying activation) and modifying APD, and so the behavior of re-entry was found to depend on the configuration of heterogeneities. Figure 9 illustrates different wavelengths by showing activation isochrones during one cycle of re-entry in a simulation with the *CRNnormal* variant (Figure 9A), and one cycle in a simulation with the *CRNremodelled* variant. Both simulations had a length scale of 10.0 mm, and the heterogeneity in diffusion was identical. In both cases, re-entry propagates clockwise around an inexcitable region in the top left quadrant of the simulated tissue, and this is an example of re-entry “pinned” to a heterogeneity (Zahid et al., 2016; Deng et al., 2017). The activation pathway is illustrated by a blue arrow, and the wavelength is the distance between the wavefront (white regions), and the wave back (dark red). The short wavelength associated with the shorter APD in the *CRNremodelled* variant results in a compact re-entrant pathway (Figure 9), whereas the longer wavelength associated with the *CRNnormal* variant results in an extended re-entrant pathway (Figure 9A), and a much longer period of re-entry. Movies of these simulations are available as Videos 5, 6.

## 4. Discussion and conclusions

It is well-known that electrical heterogeneity is an important factor for determining vulnerability to arrhythmias in cardiac tissue, and this has been investigated in experimental preparations (Allessie et al., 1976; Gokhale et al., 2017), in intact atria (Haissaguerre et al., 2016), and in computer models (Xie et al., 2001; Vigmond et al., 2004; Clayton and Holden, 2005; McDowell et al., 2015; Kazbanov et al., 2016; Morgan et al., 2016). Fibrosis is believed to associated with atrial fibrillation in the human heart, and regions of fibrosis can be focal or diffuse (McDowell et al., 2015). The present study investigated how heterogenous diffusion varying on different length scales influenced electrical activation, electrical recovery, APD, vulnerability to re-entry, and dynamical behavior of re-entry in a model of human atrial tissue. Regions of concentrated fibrosis were represented by coupled but inexcitable tissue. The main findings of the study are as follows:

- It is possible to represent heterogeneity in cardiac tissue conductivity at different length scales using samples of a GRF.
- The length scale of heterogeneity did not markedly affect the median delay in activation time relative to simulations with a uniform diffusion coefficient of 0.2 mm^2^ms^−1^, but larger length scales of 5.0 and 10.0 mm tended to produce a wider spread of activation delays.
- A delay in recovery relative to simulations with a uniform diffusion coefficient was found. This tended to be greater in simulations with heterogeneity on a smaller length scale, and greater with the *CRNnormal* variant rather than *CRNremodelled*.
- Distributions of APD tended to be positively skewed toward higher APD with the *CRNnormal* variant and negatively skewed toward lower APD with the *CRNremodelled* variant. This difference was attributed to inexcitable regions of simulated fibrosis acting as a current source during repolarization in simulations with the *CRNnormal* variant, and as a current sink in simulations with the *CRNremodelled* variant.
- With decremental pacing, simulations with smaller length scale heterogeneity and the *CRNremodelled* variant were most likely to result in sustained re-entry. However, the configuration of regions of simulated fibrosis also played an important role.
- With a spiral wave as an initial condition, simulations with smaller length scale heterogeneity and the *CRNremodelled* variant were also most likely to result in sustained re-entry

The interaction of heterogenous diffusion, length scale, simulated fibrosis, and APD is complex and merits further investigation. The relationship of this study to previous work is discussed below.

### 4.1. Model of fibrosis

The findings of the present study depend on the way that cellular electrophysiology and regions of fibrosis have been represented. In previous studies, regions of fibrosis have been represented in many different ways including inexcitable obstacles (Ten Tusscher and Panfilov, 2007), coupled elements with a fixed resting potential (Majumder et al., 2012), and with detailed fibroblast models (Sachse et al., 2009; Ashihara et al., 2012; Morgan et al., 2016; Zahid et al., 2016). The extent of coupling between fibroblasts and myocytes in the heart remains controversial because detailed experimental study of fibroblasts embedded in native cardiac tissue is difficult (Kohl and Gourdie, 2014), but novel experimental techniques have shown that non-myocytes undergo voltage excursions close to the border of scar tissue (Quinn et al., 2016). Regions of fibrosis have often been represented as areas of reduced tissue conductivity or a lower diffusion coefficient in computational studies (Gonzales et al., 2014; McDowell et al., 2015; Zahid et al., 2016).

In the present study regions of diffuse fibrosis were represented by smoothly varying but reduced diffusion, and focal fibrosis was represented as inexcitable tissue with a fixed diffusion coefficient of 0.05 mm^2^ms^−1^. The slowed upstroke and lower amplitude of voltage excursion observed in simulated fibrosed regions compared to normal tissue was comparable to other studies that have used more detailed models of fibroblast electrophysiology (Ashihara et al., 2012; Zahid et al., 2016) as well as experimental observations (Kohl and Gourdie, 2014). Nevertheless, further work with more detailed fibroblast models would be a valuable extension to the present study.

An additional set of simulations was done to compare the effect of simulations that used smoothly varying diffusion fields with simulations where the diffusion coefficient was set to 0.05 mm^2^ms^−1^ in fibrosed regions and 0.2 mm^2^ms^−1^ elsewhere (see Supplementary Figure A). With the *CRNnormal* variant, simulations with abrupt changes in diffusion had a longer APD and greater APD dispersion, especially at shorter length scales, compared to simulations with smoothly varying diffusion. With both variants, the delay in activation time was much shorter compared to simulations with smoothly varying diffusion. The overall response to decremental pacing was also similar for the *CRNnormal* variant, but it was not possible to elicit more than one additional beat with the *CRNremodelled* variant (see Supplementary Figure B). These findings indicate that there are important differences in the behavior of simulated tissue with abrupt and smooth changes in diffusion coefficient.

### 4.2. Effect of length scale

The length scale of heterogeneity was found to play a role in the prolongation and dispersion of APD, as well as in determining vulnerability to re-entry and rotor stability. A shorter length scale favored a longer APD, greater APD dispersion, and sustained re-entry. The configuration of simulated fibrotic regions was also important because a small isthmus of tissue often formed part of the activation pathway during re-entry as illustrated in Figure 7. Images of human left atria show fibrosis on a range of different length scales (McDowell et al., 2015), and the observations in the present study are consistent with result from patient specific models, where configuration of fibrotic regions was found to determine rotor location and stability (Zahid et al., 2016; Deng et al., 2017).

One of the aims of the present study was to investigate the effect of smooth changes in diffusion. Since diffusion was capped at 0.2 and 0.05 mm^2^ms^−1^, there were discontinuities at the boundary of these regions as illustrated in Figures 1E,F. While no obvious numerical problems were identified, a recent study indicates that numerical approximation of a continuum model may not capture the biophysics of heterogenous tissue fully (Gokhale et al., 2017). The conduction velocity observed with a uniform diffusion coefficient (Figure 2A) in the present study was lower than those observed clinically (Weber et al., 2011), with an expected decrease in conduction velocity associated with fibrosis manifest as an activation delay (Figure 3A). For future work capping at a higher diffusion coefficient may be needed to better match conduction velocity, and this could potentially introduce numerical problems.

### 4.3. Effect of AF-induced remodeling

Persistent or permanent AF leads to changes in the electrophysiology of atrial myocytes, which act to shorten APD and hence wavelength, and these changes are consistent with the observed progression of AF from a short-lived paroxysmal arrhythmia to a permanent state (Nattel, 2002; Colman et al., 2013). In the present study, two variants of the CRN model were used, *CRNnormal* representing normal atrial myocytes, and *CRNremodelled* representing remodeled atrial myocytes (Wilhelms et al., 2012). The shorter APD of the *CRNremodelled* variant tended to modulate the effects of inexcitable fibrotic regions, and to increase vulnerability to re-entry. A further consequence of the shorter APD was the shorter wavelength of the *CRNremodelled* variant, which favored sustained re-entry in almost all simulations with the *CRNremodelled* variant and a spiral wave as an initial condition (Figure 8).

It is possible that more complex effects come into play in real cardiac tissue. Isolated atrial myocytes from canine right atrium show a wide range of action potential shapes (Feng et al., 1998), and the variability of APD measured in human atrial cells indicate that a similar range of shape is likely in the human heart (Sánchez et al., 2014). A further consideration is changes in the Ca^2+^ handling mechanisms, which are affected by remodeling and also feed back into action potential shape (Grandi et al., 2011). These effects are likely to be important because in the present study, local source and sink effects acted to modify action potential shape in the absence of underlying cellular heterogeneity (Figure 5A).

### 4.4. Insights into AF mechanisms

The anatomy and configuration of fibrosis has recently been shown to be more important in determining rotor location than cellular electrophysiology (Deng et al., 2017). In the present study there were strong indications that the configuration of fibrosed regions, combined with length scale and electrical remodeling, acted together to determine APD dispersion and arrhythmogenic potential. Since configuration was found to be important, there was not a simple association between APD dispersion and vulnerability to re-entry. Extending this study to detailed anatomical representations of the human atria (Morgan et al., 2016; Varela et al., 2016) would begin to shed light on the underlying principles. While there is progress toward building patient specific models of human atria based on imaging of fibrosis as tools to guide therapy (Trayanova et al., 2014; Morgan et al., 2016), the present study indicates that there is more to learn about the way that electrical activation and recovery are modulated by heterogeneous diffusion. A better understanding of this complexity may in the future lead to improvements in our understanding of the factors that favor and sustain AF in some individuals and not others.

### 4.5. Study limitations

This study used a very simple representation of fibrosis, as discussed above. There was no attempt to represent the detailed anatomy of the human atria, which would add a further layer of complexity to the findings. In particular, diffusion was assumed to be isotropic, although fibrosis is known to have a stronger effect on lateral connections between myocytes, acting to increase anisotropy (Kohl and Gourdie, 2014). In the human atria, the action potential acts to initiate and synchronize mechanical contraction, and fibrosis would be expected to alter the mechanical properties of atrial tissue. There was no attempt to include atrial mechanics in the present study, and heterogeneous contraction may add a further layer of complexity to the understanding of fibrosis and atrial arrhythmogenesis. Despite these limitations, this investigation has demonstrated a novel application of GRFs to represent heterogeneous diffusion at different length scales in cardiac tissue.

## Author contributions

RC designed the study, wrote and implemented the computational model, ran the simulations, prepared the figures, and wrote the manuscript.

### Conflict of interest statement

The author declares that the research was conducted in the absence of any commercial or financial relationships that could be construed as a potential conflict of interest.

## Supplementary material

Supplementary Figure A Image modified version of Figure 3, which includes results from simulations with abrupt transitions between normal and fibrotic regions.

Supplementary Figure B Image modified version of Figure 6, which includes results from simulations with abrupt transitions between normal and fibrotic regions.

Video 1 Video shows a movie of rapid pacing resulting in sustained re-entry in a simulation with a length scale of 2.5 mm and the *CRNnormal* model variant, corresponding to Figures 7A,B.

Video 2 Video shows a simulation with a length scale of 5 mm and the *CRNremodelled* model variant, corresponding to Figures 7C,D.

Video 3 Video shows a further example where rapid pacing results in sustained re-entry in a simulation with a length scale of 2.5 mm and the *CRNremodelled* model variant.

Video 4 Video shows non-sustained re-entry following initiation with a spiral wave in a simulation with a length scale of 1.25 mm and the *CRNnormal* model variant.

Video 5 Video shows sustained re-entry following initiation with a spiral wave in a simulation with a length scale of 10 mm and the *CRNnormal* model variant, corresponding to Figure 9A.

Video 6 Video shows sustained re-entry following initiation with a spiral wave in a simulation with a length scale of 10 mm and the *CRNremodelled* model variant, corresponding to Figure 9B.
