# Supplementary figures and images for: Dispersion of Recovery and Vulnerability to Re-entry in a Model of Human Atrial Tissue With Simulated Diffuse and Focal Patterns of Fibrosis
Source: Front Physiol. 2018 Aug 7;9:1052. doi: 10.3389/fphys.2018.01052 (PMC6090998; doi:10.3389/fphys.2018.01052)

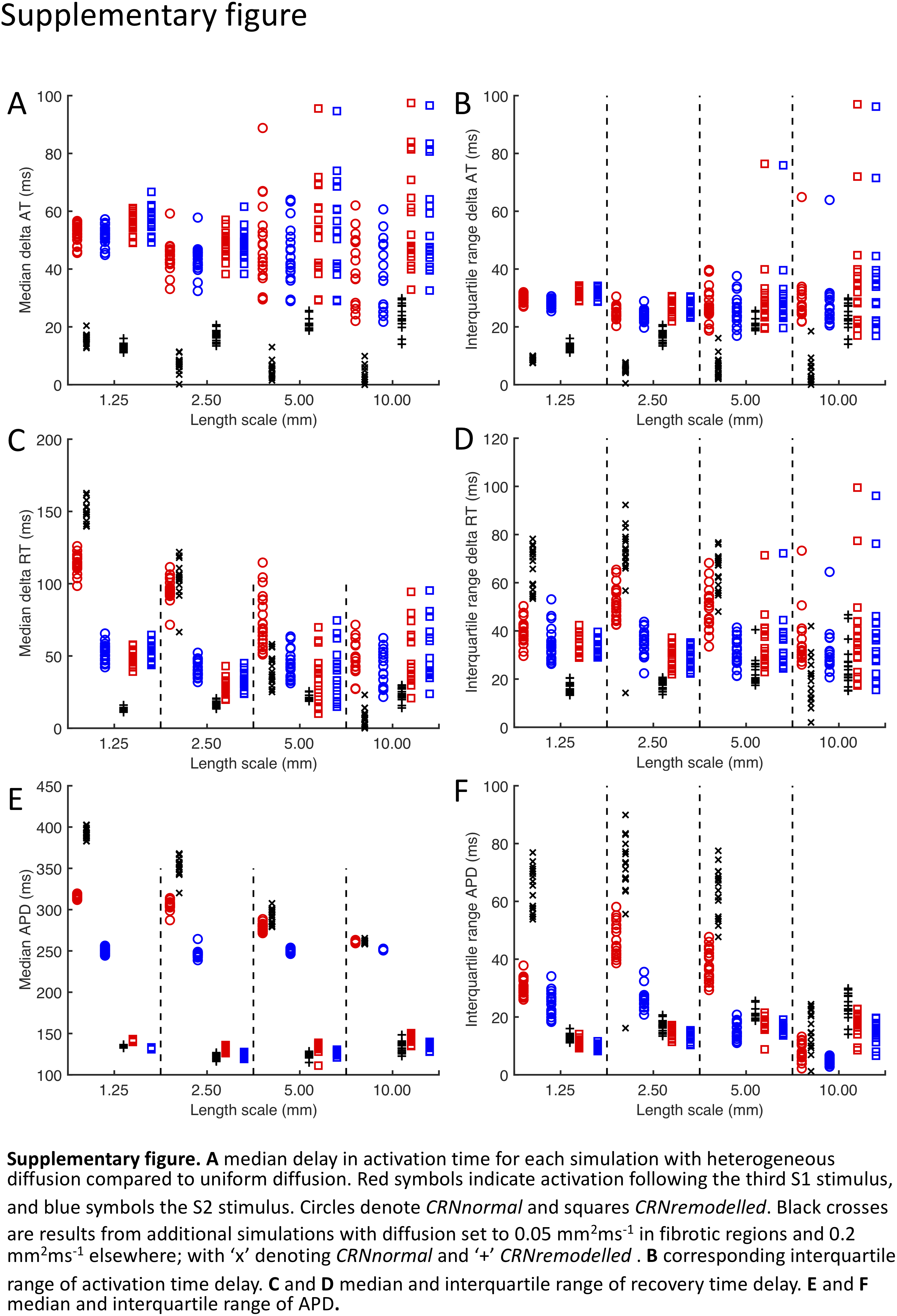

Supplement: Supplementary Figure A — Image modified version of Figure 3, which includes results from simulations with abrupt transitions between normal and fibrotic regions. [file Image_1.TIFF]

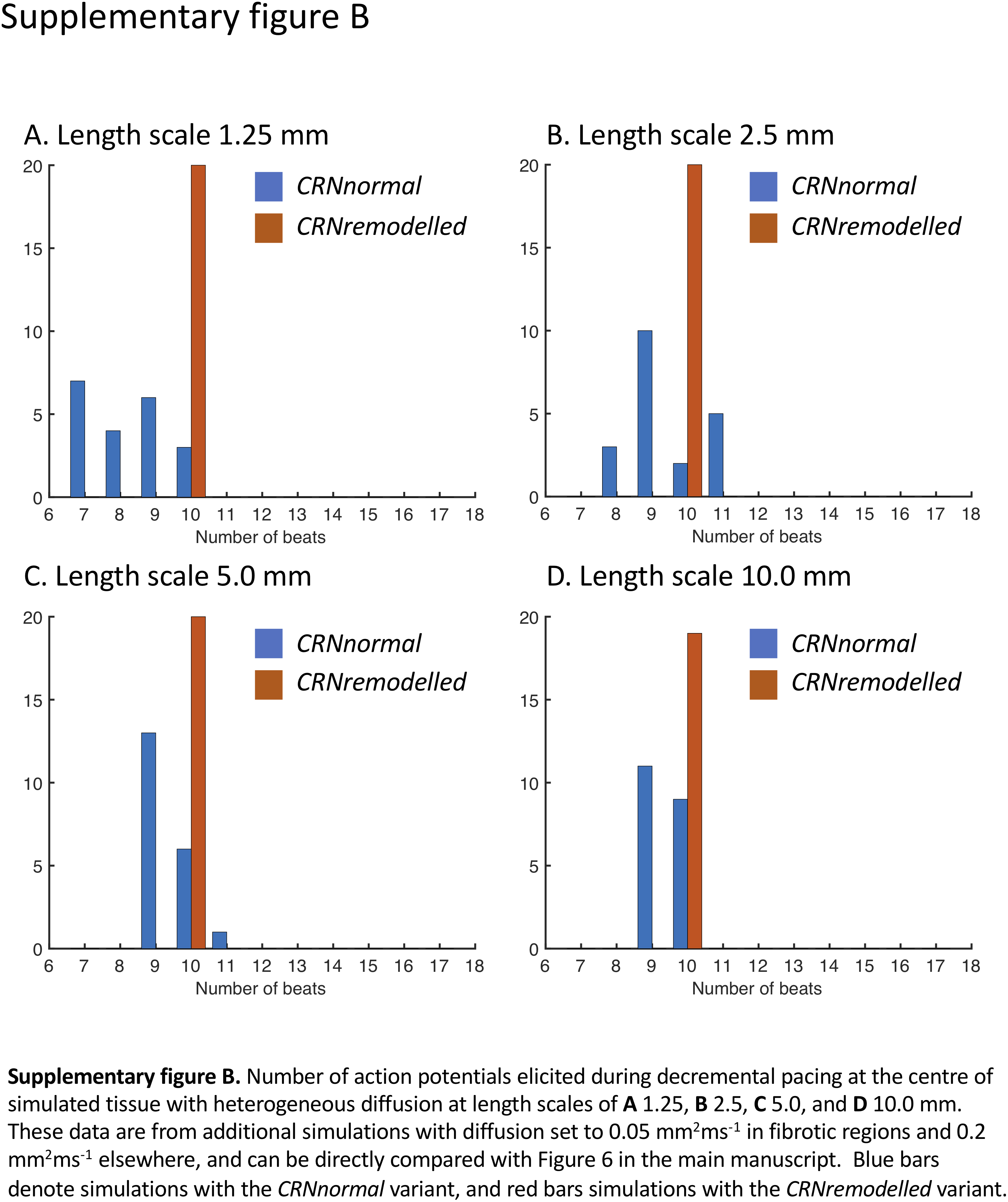

Supplement: Supplementary Figure B — Image modified version of Figure 6, which includes results from simulations with abrupt transitions between normal and fibrotic regions. [file Image_2.TIFF]
